# Supplementary material for: Organelle Volume Facilitates Spindle Positioning During C. elegans Female Meiosis
Source: bioRxiv. 2026 Oct 9:2026.10.07.757430. Preprint. [Version 1] doi: 10.64898/2026.10.07.757430 (PMC13692239; doi:10.64898/2026.10.07.757430)
Supplement: Supplementary file 1 [file NIHPP2026.10.07.757430V1-supplement-1.pdf]

bioRxiv preprint doi: https://doi.org/10.64898/2026.10.07.757430; this version posted October 9, 2026. The copyright holder for this preprint (which was not certified by peer review) is the author/funder, who has granted bioRxiv a license to display the preprint in perpetuity. It is made available under a CC-BY 4.0 International license.

Figure S1

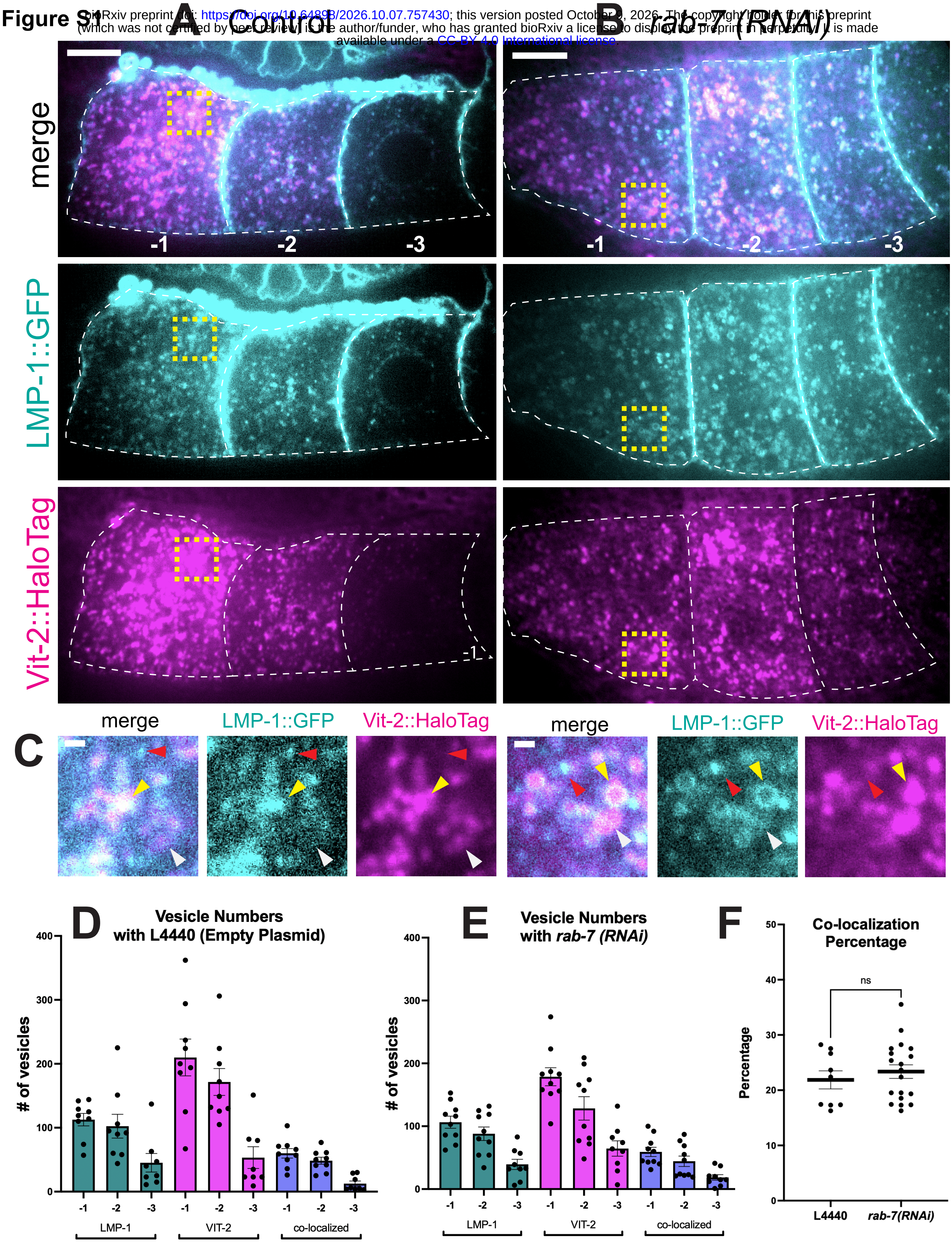

bioRxiv preprint doi: https://doi.org/10.64898/2026.10.07.757430; this version posted October 9, 2026. The copyright holder for this preprint (which was not certified by peer review) is the author/funder, who has granted bioRxiv a license to display the preprint in perpetuity. It is made available under a CC-BY 4.0 International license.

Figure S1: Vitellogenin co-localization with lysosomes

(A-B) Single focal plane fluorescence images of a living young adult worm germline from the –1 to the –3 oocyte. LMP-1::GFP in cyan and VIT-2::HALO in magenta. (A) Control condition where worms were fed with L4440 (empty plasmid). (B) Worms treated with *rab-7(RNAi)* have artificially enlarged late endosomes to better visualize co-localization on the surface of vesicles. Scale Bar = 10µm

(C) Enlarged images from yellow dashed squares in Fig. S1A, Fig.S1B showing vesicle localization and co-localization between lysosomal protein (LMP-1) and VIT-2. Yellow arrowhead = co-localized vesicles. Red arrowhead = LMP-1 only. White arrowhead = VIT-2 only. Scale Bar = 1µm

(D-E) Graph showing the numbers of LMP-1, VIT-2, and co-localized vesicles across the germline (-1 through -3) in the control (D) and *rab-7(RNAi)* (E) condition.

(F) Graph of the co-localization percentage of vesicles in both conditions. L4440: Mean: 21.87% ± 1.63; *rab-7(RNAi):* Mean: 23.36% ± 1.22

bioRxiv preprint doi: https://doi.org/10.64898/2026.10.07.757430; this version posted October 9, 2026. The copyright holder for this preprint (which was not certified by peer review) is the author/funder, who has granted bioRxiv a license to display the preprint in perpetuity. It is made available under a CC-BY 4.0 International license.

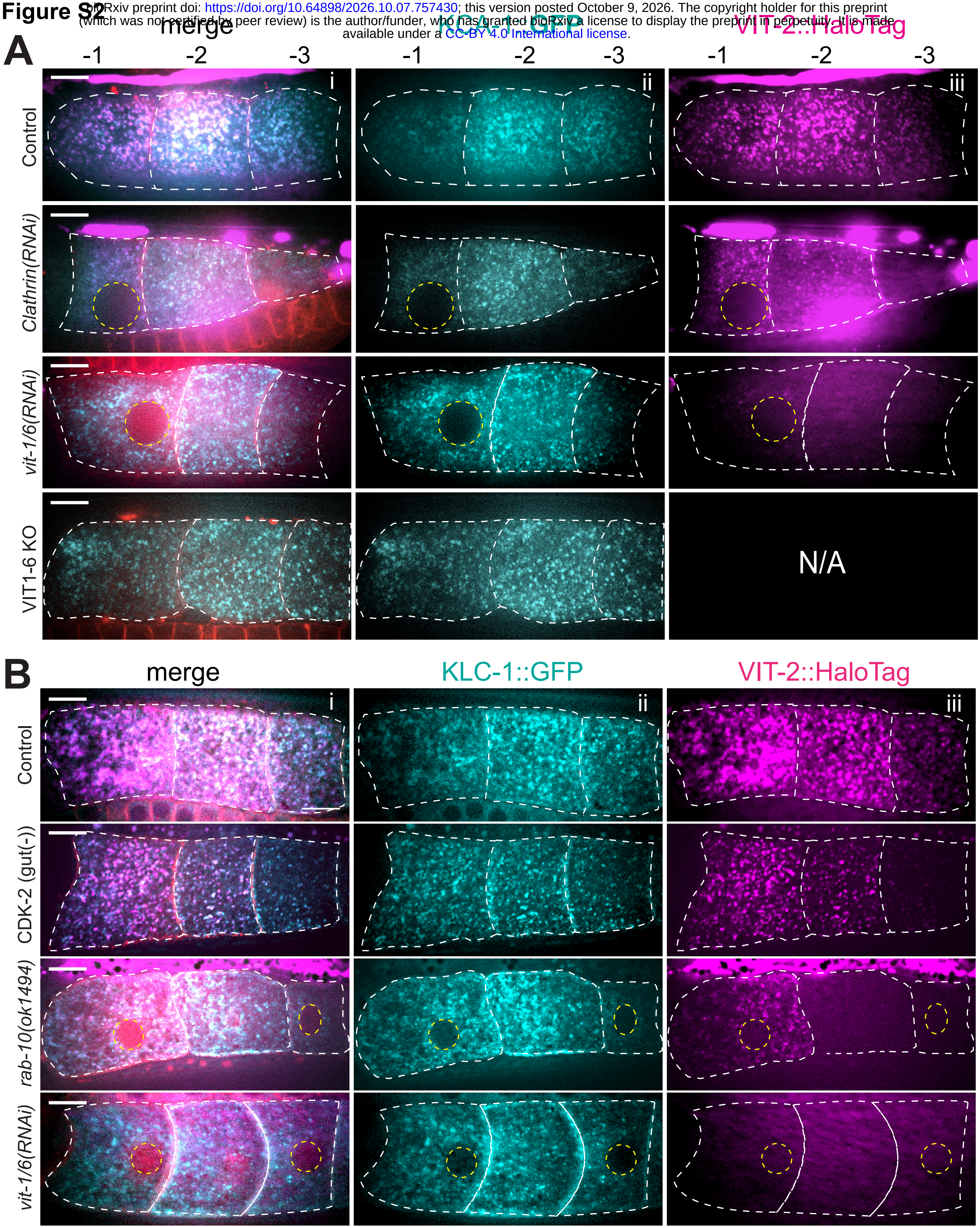

bioRxiv preprint doi: https://doi.org/10.64898/2026.10.07.757430; this version posted October 9, 2026. The copyright holder for this preprint (which was not certified by peer review) is the author/funder, who has granted bioRxiv a license to display the preprint in perpetuity. It is made available under a CC-BY 4.0 International license.

Figure S2: Vitellogenin Depletion Strain Gonads

(A-B) Single focal plane fluorescence images of a living young adult worm germline from the –1 to the –3 oocyte in strains and conditions with vitellogenin depletion. Some oocytes do not contain a nucleus because images are taken near the top Z-plane for better vesicle resolution. (i) Merge of fluorescence channels. (ii) Green (488 excitation) showing endogenously tagged KCA-1::GFP (A) or KLC-1::GFP (B). (iii) Magenta (640 excitation) showing endogenously tagged VIT-2::HaloTag labelled with JaneliaFluor646 HaloTag ligand. Scale bar = 10μm. White dashed line = plasma membrane of oocytes. Yellow dashed circles = nucleus outline.

bioRxiv preprint doi: https://doi.org/10.64898/2026.10.07.757430; this version posted October 9, 2026. The copyright holder for this preprint (which was not certified by peer review) is the author/funder, who has granted bioRxiv a license to display the preprint in perpetuity. It is made available under a CC-BY 4.0 International license.

Figure S3

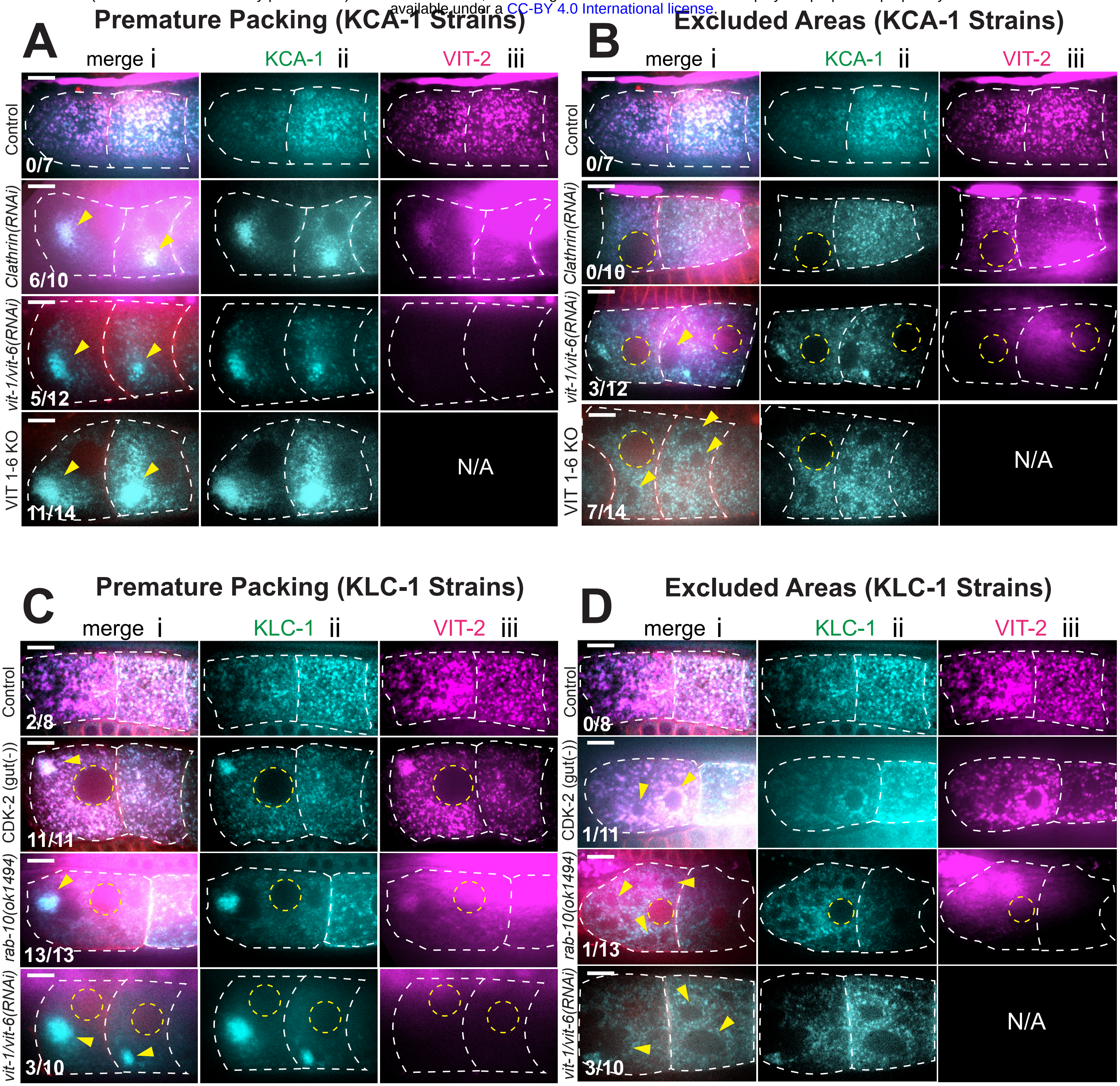

bioRxiv preprint doi: https://doi.org/10.64898/2026.10.07.757430; this version posted October 9, 2026. The copyright holder for this preprint (which was not certified by peer review) is the author/funder, who has granted bioRxiv a license to display the preprint in perpetuity. It is made available under a CC-BY 4.0 International license.

Figure S3: Vitellogenin Depletion Phenotypes

(A-B) Single focal plane fluorescence images of a living young adult worm germline from the –1 to the –2 oocyte in strains and conditions with vitellogenin depletion. (i) Merge of fluorescence channels. (ii) Green (488 excitation) showing endogenously tagged KCA-1::GFP (iii) Magenta (640 excitation) showing endogenously tagged VIT-2::HaloTag labeled with JaneliaFluor646 HaloTag ligand. Scale bar = 10μm. White dashed line = plasma membrane of oocytes. Yellow dashed circles = nucleus outline. Yellow arrows: premature packing (A) and regions of exclusion (B).

(C-D) Single focal fluorescence images of a living young adult worm germline from the –1 to the –2 oocyte in strains and conditions with vitellogenin depletion. (i) Merge of fluorescence channels. (ii) Green (488 excitation) showing endogenously tagged KLC-1::GFP (iii) Magenta (640 excitation) showing endogenously tagged VIT-2::HaloTag labeled with JaneliaFluor646 HaloTag ligand. Scale bar = 10μm. White dashed line = plasma membrane of oocytes. Yellow dashed circles = nucleus outline. Yellow arrows: premature packing (C) and regions of exclusion (D).
